# On the Chinese species of the genus *Intestinarius* Kurbatov (Coleoptera, Staphylinidae, Pselaphinae)

**DOI:** 10.3897/zookeys.116.1329

**Published:** 2011-07-07

**Authors:** Zi-Wei Yin, Li-Zhen Li, Mei-Jun Zhao

**Affiliations:** Department of Biology, College of Life and Environmental Sciences, Shanghai Normal University, Shanghai, 200234, P. R. China

**Keywords:** Coleoptera, Staphylinidae, Pselaphinae, *Intestinarius*, new species, key, taxonomy, China

## Abstract

A total of three Chinese species of the genus *Intestinarius* Kurbatov, 2007 are recognized, among which, two new species are described: *Intestinarius guangdongensis* **sp. n.** from Guangdongand *Intestinarius  longiceps* **sp. n.** from Guizhou. Illustrations of major diagnostic characters are given for all three Chinese species including *Intestinarius kuzmini* Kurbatov, 2007. A key is included to aid in their identification.

## Introduction

Eight species of the genus *Intestinarius* Kurbatov, 2007 (type species: *Batrisodes quinquesulcatus* Raffray, 1904) have been so far known from the Southeast Asia: one from Indonesia, two from Laos, three from Malaysia, one from both Singapore and Malaysia and one from China. *Intestinarius* was thought to be closely allied to *Mnia* Newton & Chandler, 1989 by certain shared morphological characters (Kurbatov 2007: 282; referring to *Mnia* see Löbl (1973)) and may be distinguished from *Mnia* by the presence of well-defined longitudinal sulci on the head and pronotum, by the first antennomere not remarkably elongate, by each elytron with three basal foveae, and by the sternite IV without discal carinae (basolateral incisions in Kurbatov (2007)).

Species of *Intestinarius* are morphologically similar and the correct identification must be based on the study of male sexual characters. In the present study, we found that characters on female genitalia may also provide a faithful identification.

During several recent collecting trips conducted in South and Southwest China, the authors and their colleagues collected some *Intestinarius* specimens in Guangdong, Guizhou, and Yunnan Provinces which prove to be two new and one known species. The purpose of this paper is to describe the new species, to provide illustrations of major diagnostic characters and a key for the identification of the so far known Chinese species.

## Material and methods

All specimens were collected from the leaf litter of the forest floor by sifting. They were killed with ethylacetate and then dried. Dissections were done in 75% ethanol. The genital organs and other dissected parts were mounted in Euparal (Chroma Geselschaft Schmidt, Koengen, Germany) on plastic slides that were placed on the same pin as the specimen. Photos were taken by a Canon EOS 40D Camera mounted with an MP-E 65 mm Macro Photo Lens or by a Canon G9 Camera mounted on an Olympus CX31 microscope; line drawings were made using Adobe Illustrator CS2.

Slash (/) is used to separate different lines on the same label. The terminology of foveal system follows Chandler (2001).

The following acronyms are used in the text:

BL	length of body (= hl+pl+el+al)

HL	maximum length of head, measured from anterior margin of clypeus to posterior base, excluding occiput

HW	maximum width of head, measured across eyes

PL	length of pronotum, measured along midline

PW	maximum with of pronotum

EL	length of elytra, measured along sutural line

EW	maximum width of elytra

AL	maximum length of abdomen

AW	maximum width of abdomen.

Measurements were made based on a random sample of 45 specimens, unite is in millimeter.

All specimens studied are deposited in the Insect Collection of Shanghai Normal University, Shanghai, China (SNUC).

## Taxonomy

### Intestinarius guangdongensis

Yin and Li sp. n.

urn:lsid:zoobank.org:act:2AA8965E-E7DA-4B7F-BA3B-1344CF1EB95B

http://species-id.net/wiki/Intestinarius_guangdongensis

**Figures 1–2.**
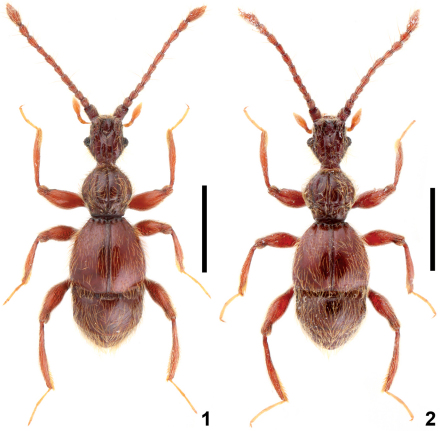
Male habitus of *Intestinarius*spp. **1** *Intestinarius guangdongensis***2** *Intestinarius  longiceps*. Scales: 1.0 mm.

Figs 1 3 6, 9, 12 15–22 39–40, 45–46 

#### Type locality.

Nan-ling National Nature Reserve, Guangdong Province, South China

#### Type material

(7 ♂♂, 5 ♀♀)**.** Holotype: ♂, labelled ‘**CHINA:** guangdong Prov. / Shaoguan City / Nanling National N. R. / 09.viii.2010 / Li-Zhen LI leg.’; Paratypes: 6 ♂♂, 5 ♀♀, same label data as holotype.

#### Description.

 Measurements: male (female). BL 2.62–2.72 (2.40–2.56), HL 0.58–0.63 (0.58–0.60), HW 0.52–0.55 (0.49–0.50), PL 0.56–0.58 (0.55–0.56), PW 0.58–0.59 (0.53–0.57), EL 0.87–0.89 (0.77–0.78), EW 0.93–0.94 (0.84–0.88), AL 0.61–0.62 (0.50–0.62), AW 0.83–0.85 (0.80–0.81). Length of aedeagus 0.41. Width of female genitalia 0.32.

**Figures 3–5.**
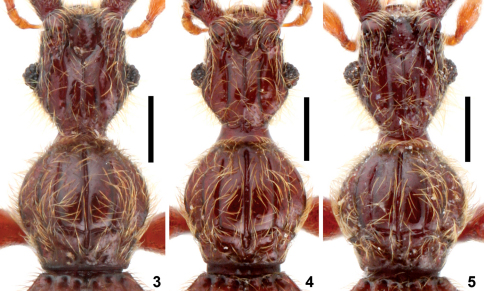
Head and pronotum of *Intestinarius*spp., male. **3** *Intestinarius guangdongensis***4** *Intestinarius kuzmini* **5** *Intestinarius  longiceps*. Scales: 0.3 mm.

**Figures 6–14.**
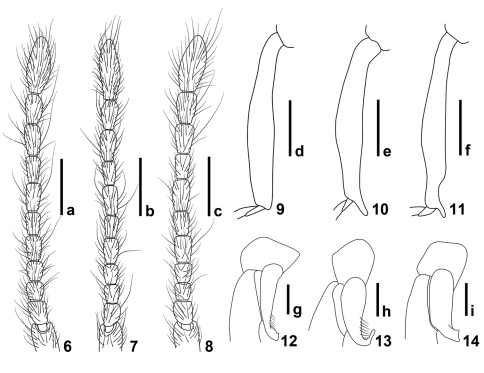
Details of *Intestinarius*spp., male. **6–8** antenna **9–11** mesotibia **12–14** metatrochanter **6, 9, 12** *Intestinarius guangdongensis***7, 10, 13** *Intestinarius kuzmini* **8, 11, 14** *Intestinarius longiceps*. Scales: a–c = 0.3 mm, d–f = 0.2 mm, g–i = 0.1 mm.

Male (Fig. 1). Reddish-brown, maxillary palpi and tarsi lighter. Head, pronotum and elytra covered with long setae. Head (Fig. 3) slightly longer than wide. Clypeus short in dorsal view, roundly arcuate on anterior margin; frons markedly impressed between profoundly raised antennal tubercles; vertexal foveae situated backwards, vertexal sulci extended from vertexal foveae to frons; carinae lateral to the vertexal sulci derived from base of antennal tubercles well-defined; occipital carina reaching posterior margin of frontal impression; postocular margins moderately long, nearly straight, gradually narrowed toward base. Eyes well-developed, each comprised of about 25 facets. Fourth palpomere of maxillary palpi with tuft of dense and fine external setae. Gular carina not conjoint with gular fovea. Antennae as in Fig. 6. Pronotum (Fig. 3) about as long as wide, median longitudinal sulcus extended beyond transverse antebasal sulcus, two lateral longitudinal sulci well-defined, exterior one born from lateral antebasal foveae; two pairs of basolateral foveae and two pairs of antebasal conical tubercles well-defined. Each elytron with three basal foveae and complete sutural stria; discal stria short, reaching less than half of elytral length. Mesotrochanters slightly protuberant on posterior margin, mesotibiae without apical protuberance; metatrochanters modified (Fig. 12), setose on posterior margin.

**Figures 15–22.**
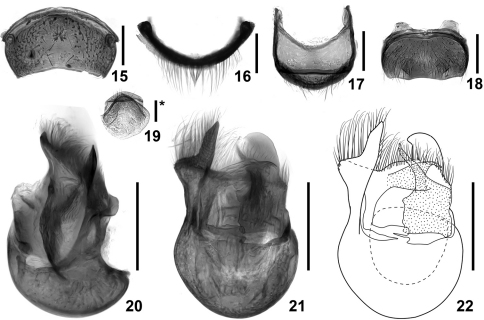
Details of *Intestinarius guangdongensis*, male. **15** tergite VII **16** same, anterior view **17** tergite VIII **18** sternite VIII **19** sternite IX **20** aedeagus, lateral view **21–22** same, dorsal view. Scales: 0.2 mm (scale with ‘*’ = 0.1 mm).

Abdomen with tergite IV largest, V–VI about same length and successively narrowed, tergite (Figs 15–16) VII modified, provided with median small round impression, tuft of long setae directed toward posterior margin; tergite VIII as in Fig. 17; sternite VIII as in Fig. 18; sternite IX as in Fig. 19; aedeagus as in Figs 20–22.

Female. Slightly smaller than male; each eye comprised of about 15 facets; maxillary palpi, legs and tergite VII lacking modifications; tergite VIII as in Fig. 39; sternite VIII as in Fig. 40; female genitalia as in Figs 45–46.

**Distribution.** Known only from the type locality.

**Etymology.** The species is named after its type locality.

**Ramarks.** The modified maxillary palpomere IV of the new species is shared only with *Intestinarius quinquesulcatus*, type species of the genus, described from Singapore and Maylasia; the unmodified male mesotibiae is similar to that of *Intestinarius orthopygium* (Laos); the modified metatrochanters resemble those of *Intestinarius distorticeps* (Jawa), *Intestinarius quinquesulcatus*, *Intestinarius kuzmini* (Yunnan, China), *Intestinarius orthopygium*, *Intestinarius crassicornis* (Laos) and *Intestinarius longiceps* sp. n. (Guizhou, China); the modified tergite VII also occurs in most species of the genus except for *Intestinarius distorticeps* and *Intestinarius pexatus*. *Intestinarius guangdongensis* may be dintinguished from all its congeners by a combination of the following characters: simple mesotibiae, shape of modified metatrochanters and shape of sternite VIII in male, shape of female tergite and sternite VIII and genital organs of both sexes.

One female specimen has tergite VII with median protuberance, but it is identical to other females in all characters including female genitalia. Thus, females of this species are most probably polymorphic in this character. Such polymorphism in *Intestinarius kuzmini* was also observed in Kurbatov (2007: 284) and in the present study (see remarks under *Intestinarius kuzmini*).

### Intestinarius kuzmini

Kurbatov

http://species-id.net/wiki/Intestinarius_kuzmini

Figs 4 7, 10, 13 23–30 41–42, 47–48

Intestinarius kuzmini  Kurbatov, 2007: 283

#### Type locality.

Mengyang Nature Reserve, South Yunnan Province, Southwest China.

#### Material studied

(13 ♂♂, 9 ♀♀)**.** 4 ♂♂ (1 ♂ with only aedeagus remained in Euparal), 2 ♀♀, labelled ‘**CHINA:** yunnan Prov. / Na-Ban-He N. R. / Xiao-nuo-you-xia-zhai / 20.ix.2008, alt. 950 m / HU & TANG leg.’; 5 ♂♂, 5 ♀♀, same, but ‘9.ix.2008, alt. 1,500 m’; 3 ♂♂, same, but ‘Man-fei / 18.ix.2008, alt. ca. 600 m’; 1 ♀, same, but ‘Guo-men-shan / 21.xi.2008, alt. ca 1,000 m / HU & TANG leg.’; 1 ♂, same, but ’08.v.2009, alt. 1,200 m / HU & YIN leg.’; 1 ♀, same, but ‘05.i.2004 / LI & TANG leg.’.

#### Description.

Measurements: male (female). BL 2.55–2.61 (2.42–2.57), HL 0.56–0.58 (0.55–0.56), HW 0.51–0.52 (0.50–0.51), PL 0.57–0.58 (0.56–0.57), PW 0.56–0.57 (0.55–0.56), EL 0.81–0.82 (0.73–0.75), EW 0.89–0.92 (0.90–0.92), AL 0.61–0.63 (0.58–0.69), AW 0.83–0.85 (0.86–0.88). Length of aedeagus 0.52. Width of female genitalia 0.28.

Eyes of male comprised of about 25 facets, of female of about 15 facets.

Detailed description of *Intestinarius kuzmini* refers to Kurbatov 2007: 283–284. Additional illustrations of the following major diagnostic characters are provided. Male: head and pronotum (Fig. 4), left antenna (Fig. 7), modified mesotibia (Fig. 10) and metatrochanter (Fig. 13), tergite VII (Fig. 23–24), tergite VIII (Fig. 25), sternite VIII (Fig. 26), sternite IX (Fig. 27) and aedeagus (Figs 28–30). Female: tergite VIII (Fig. 41), sternite VIII (Fig. 42) and genitalia (Figs 47–48).

**Figures 23–30.**
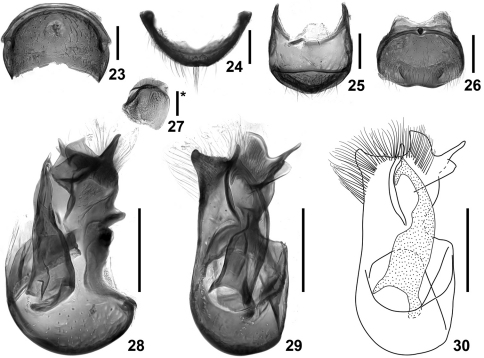
Details of *Intestinarius kuzmini*, male. **23** tergite VII **24** same, anterior view **25** tergite VIII **26** sternite VIII **27** sternite IX **28** aedeagus, lateral view **29–30** same, dorsal view. Scales: 0.2 mm (scale with ‘*’ = 0.1 mm).

**Figures 31–38.**
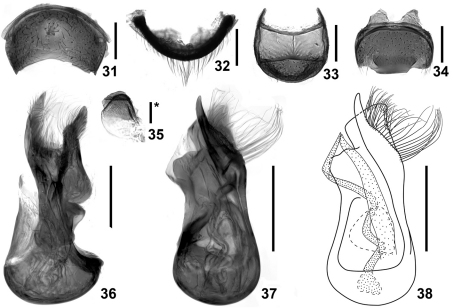
Details of *Intestinarius longiceps*, male. **31** tergite VII **32** same, anterior view **33** tergite VIII **34** sternite VIII **35** sternite IX **36** aedeagus, lateral view **37–38** same, dorsal view. Scales: 0.2 mm (scale with ‘*’ = 0.1 mm).

#### Distribution.

Known from Meng-yang and Na-ban-he Nature Reserves of Yunnan Province, Southwest China.

#### Remarks.

*Intestinarius kuzmini* is similar to its Chinese congeners with respect to the sexually modified tergite VII and metatrochanter. Male of this species may be distinguished by having long apical tooth on mesotibiae, by the shape of modified metatrochanter and by the shape of aedeagus; females may be identified based on the shape of tergite VIII, sternite VIII and genitalia.

Seven females out of nine studied have tergite VII with short, blunt to long, sharp median protuberance, but all morphological characters including genitalia are identical also with those two specimens lacking modified tergite VII. Thus, we believe that in some species of *Intestinarius*, females are most probably polymorphic in this character. (see also remarks under *Intestinarius guangdongensis*).

### Intestinarius longiceps

Yin and Li sp. n.

urn:lsid:zoobank.org:act:A6A889F4-792F-4C68-B0C3-83EACD3884DA

http://species-id.net/wiki/Intestinarius_longiceps

Figs 2 5 8, 11, 14 31–38 43–44, 49–50 

#### Type locality.

 Kuankuoshui Nature Reserve, Guizhou Province, Southwest China

#### Type material

(2 ♂♂, 10 ♀♀)**.** Holotype: ♂, labelled ‘**CHINA:** Guizhou Prov. / Kuankuoshui N. R. / Baishaogou / 04.vi.2010 / alt. 700 m / YIN & ZHAI leg.’; Paratypes: 1 ♂, 8 ♀♀, same label data as holotype; 1 ♀, same, but ‘750–900 m / 05.vi.2010 / YIN & ZHAI leg.’; 1 ♀, same, but ‘03.vi.2010 / alt. 700 m’.

#### Description.

 Measurements: male (female). BL 2.54–2.63 (2.49–2.57), HL 0.59–0.60 (0.59–0.62), HW 0.51–0.52 (0.51–0.52), PL 0.56–0.57 (0.55–0.56), PW 0.55–0.57 (0.55–0.56), EL 0.74–0.75 (0.73–0.75), EW 0.90–0.91 (0.85–0.88), AL 0.63–0.69 (0.61–0.64), AW 0.76–0.80 (0.78–0.80). Length of aedeagus 0.50. Width of female genitalia 0.27.

General morphology similar to *Intestinarius guangdongensis* sp. n., but it differs in the followings: Male maxillary palpomere IV not modified; postocular margins remarkably long; antennae (Fig. 8) more robust; mesotibiae (Fig. 11) constricted in apical portion, apical tooth slightly shorter than first tarsomere; metatrochanter (Fig. 14) on posterior margin with broad protuberance, not setose; tergite VII as in Figs 31–32; tergite VIII as in Fig. 33; sternite VIII as in Fig. 34; sternite IX as in Fig. 35; aedeagus as in Figs 36–38; female tergite VIII as in Fig. 43; sternite VIII as in Fig. 44; genitalia as in Figs 49–50.

**Figures 39–50.**
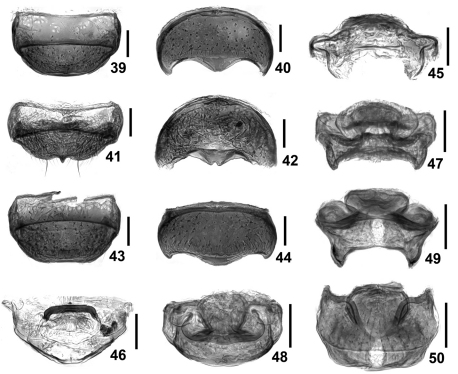
Details of *Intestinarius*spp., female. **39, 41, 43** tergite VIII **40, 42, 44** sternite VIII **45, 47, 49** female genitalia, posterior view **46, 48, 50** same, dorsal view **39, 40, 45, 46** *Intestinarius guangdongensis***41, 42, 47, 48** *Intestinarius kuzmini* **43, 44, 49, 50** *Intestinarius longiceps*.

#### Distribution.

Known only from the type locality.

#### Etymology.

 The new species is named after its long postocular margins.

#### Remarks.

 The new species may be distinguished from all its congeners by the combination of the male secondary sexual characters, *viz*. the mesotibiae and metatrochanter, the tergites VII–VIII and the sternites VIII–IX, and the shapes of male and female genitalia.

##### Key to Chinese species of the genus Intestinarius Kurbatov

All the Chinese species are externally very similar, thus, a faithful identification must be based on the dissection of the male and female genital organs combined with certain male secondary sexual characters included in the following key.

**Table:** 

1	Male: fourth maxillary palpomere with tuft of dense and short setae on exterior margin; mesotibiae lacking apical protuberance (Fig. 9); aedeagus robust, shape as in Figs 20–22. Female: sternite VIII as in fig. 40, female genitalia membranous in most parts, shape as in Figs 45–46. (Guangdong)	*Intestinarius guangdongensis* sp. n.
–	Male: maxillary palpi unmodified, mesotibiae with apical protuberance; aedeagus elongate, not fitting above. Female: sternite VIII not as in Fig. 40, female genitalia weekly sclerotized in most parts, structure not as above	2
2	Postocular margins not straight, shorter than anterior half of head (Fig. 4). Male: apical protuberance of mesotibiae much longer than first tarsomere (Fig. 10), metatrochanters on posterior margin with apically narrowed and abruptly curved thick spine, setose (Fig. 13), aedeagus as in Figs 28–30 (also see Kurbatov 2007: 284, fig. 12). Female: tergite VIII with median prominence (Fig. 41) and tergite VIII with median emargination (Fig. 42) on each posterior margin; female genitalia as in Figs 47–48. (Yunnan)	*Intestinarius kuzmini* Kurbatov
–	Postocular margins straight, about as long as anterior half of head (Fig. 5). Male: apical protuberance of mesotibiae slightly shorter than first tasomere (Fig. 11), metatrochanters on posterior margin with apically broad protuberance, lacking setae (Fig. 14), aedeagus as in Figs 36–38. Female: tergite VIII with posterior margin almost flat (Fig. 43), sternite VIII not emarginated medially (Fig. 43), female genitalia as in Figs 49–50. (Guizhou)	*Intestinarius longiceps* sp. n.

## Supplementary Material

XML Treatment for Intestinarius guangdongensis

XML Treatment for Intestinarius kuzmini

XML Treatment for Intestinarius longiceps
